# An evaluation of two types of nickel-titanium wires in terms of micromorphology and nickel ions’ release following oral environment exposure

**DOI:** 10.1186/s40510-015-0081-1

**Published:** 2015-05-20

**Authors:** Abdul Razzak A. Ghazal, Mohammad Y. Hajeer, Rabab Al-Sabbagh, Ibrahim Alghoraibi, Ahmad Aldiry

**Affiliations:** Department of Orthodontics, University of Hama Dental School, Hama, Syria; Department of Orthodontics, University of Damascus Dental School, Damascus, Syria; Nano Technology Lab, Physics Department, Faculty of Sciences, University of Damascus, Damascus, Syria; Modern Chemistry Lab, Faculty of Veterinary, University of Hama, Hama, Syria

**Keywords:** NiTi wires, Superelasticity, Heat activation, Surface morphology, Nickel release, Scanning electron microscopy, Atomic force microscopy, Atomic absorption spectrophotometer

## Abstract

**Background:**

This study aimed to compare superelastic and heat-activated nickel-titanium orthodontic wires’ surface morphology and potential release of nickel ions following exposure to oral environment conditions**.**

**Methods:**

Twenty-four 20-mm-length distal cuts of superelastic (NiTi Force I®) and 24 20-mm-length distal cuts of heat-activated (Therma-Ti Lite®) nickel-titanium wires (American Orthodontics, Sheboygan, WI, USA) were divided into two equal groups: 12 wire segments left unused and 12 segments passively exposed to oral environment for 1 month. Scanning electron microscopy and atomic force microscopy were used to analyze surface morphology of the wires which were then immersed in artificial saliva for 1 month to determine potential nickel ions’ release by means of atomic absorption spectrophotometer.

**Results:**

Heat-activated nickel-titanium (NiTi) wires were rougher than superelastic wires, and both types of wires released almost the same amount of Ni ions. After clinical exposure, more surface roughness was recorded for superelastic NiTi wires and heat-activated NiTi wires. However, retrieved superelastic NiTi wires released less Ni ions in artificial saliva after clinical exposure, and the same result was recorded regarding heat-activated wires.

**Conclusions:**

Both types of NiTi wires were obviously affected by oral environment conditions; their surface roughness significantly increased while the amount of the released Ni ions significantly declined.

## Background

Introducing nickel-titanium (NiTi) alloys has made a revolution in orthodontic wires industry [1] and made the dream of applying continuous and constant forces almost true [2]. Many improvements were applied in manufacturing austenite active (superelastic) and martensite active (heat-activated) NiTi wires to benefit from the extraordinary superelasticity and shape memory properties of NiTi alloys [3]. Unfortunately, the development of producing NiTi orthodontic wires did not prevent them from corrosion especially when exposed to corrosive conditions [4, 5].

Corrosion resistance of NiTi wires is a very important factor that affects surface roughness [6, 7] and chemical stability [8]. NiTi wires’ performance in the oral cavity has been a big concern for researchers [9–12] because it is the environment in which they are intended to function. Extreme temperatures, pH variations, and complex oral flora have made the oral environment a unique media [13] that cannot be simulated in experimental conditions [8].

Surface integrity may affect friction resistance [7]. On the other hand, instable chemical structure causes Ni ions’ release and may affect biocompatibility [14–17].

NiTi wires are usually used in the aligning and leveling stage [2], so they have to deal with complex forces that cannot be identical in all cases and vary between cases due to individual factors and the type of malocclusion.

Many researchers have evaluated and compared “as received” and “retrieved” superelastic and heat-activated NiTi wires. Those studies were performed either in unreliable circumstances (in vitro or animal studies) [2, 18–22] or involved mixed types of orthodontic NiTi wires (different manufacturer and/or composition and/or diameters) or even evaluated corrosion resistance without proper standardization of orthodontic forces (different classes of malocclusion, different inter-bracket distances, dental crown dimensions, and bone response) [6, 11, 23, 24]. Oral conditions of the in vivo studies were also not coincident, i.e., different masticatory forces, different oral hygiene status, different types of foods and drinks, mouth temperature, oral flora, and pH variations among patients [6, 10, 11, 25, 26].

Although the oral environment is a corrosive place in which superelastic and heat-activated NiTi wires are supposed to survive, it seems that there is no published data that compare between both types of NiTi wires with regard to their roughness and potential Ni ions’ release following clinical exposure in assorted in vivo conditions apart from any orthodontic forces.

## Methods

**Fig. 1:**
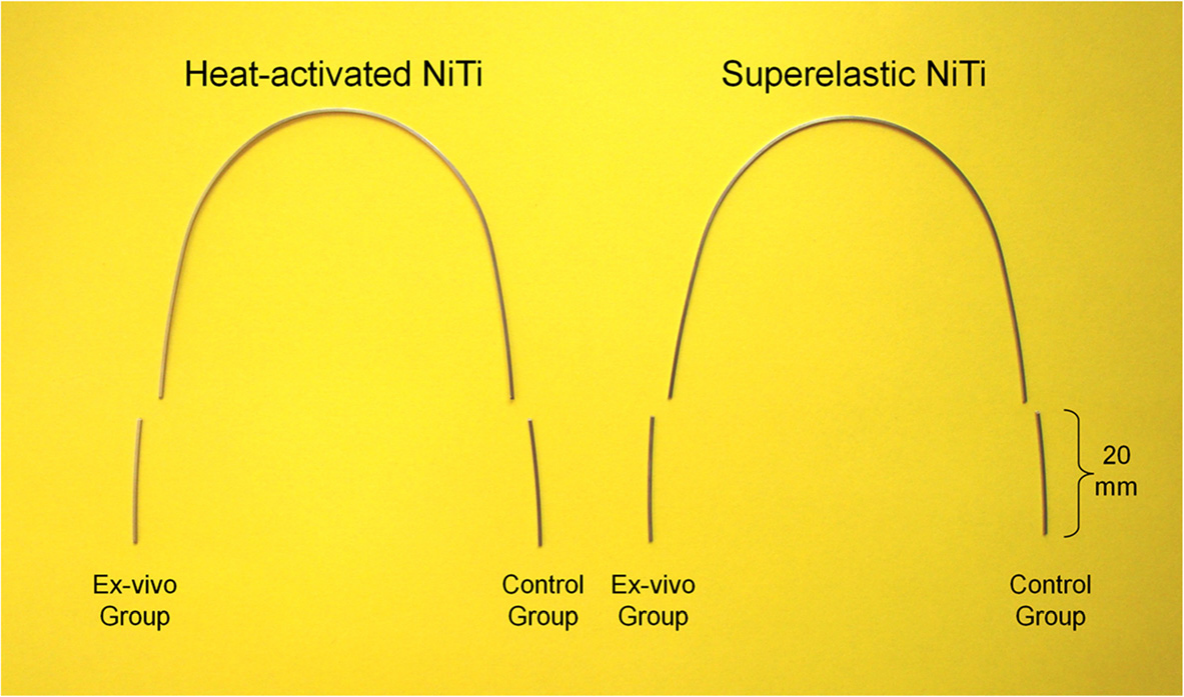
The method of constructing the control and the ex vivo wires’ groups

Two types of orthodontic nickel-titanium archwires were included: NiTi Force I (superelastic archwires) and Therma-Ti Lite (heat-activated archwires). Twelve archwires from each type were randomly selected from different packets with different batches of the same manufacturer (American Orthodontics, Sheboygan, WI, USA) and have the same chemical composition (Ni 55 %, Ti 45 %) and the same arch dimensions 0.019 × 0.025 in. Using a sterilized plier, two 20-mm-length segments of each archwire were cut from the terminal ends of both types of archwires (Fig. 1). As a result, 48 segments (24 distal cuts of NiTi Force I wires and 24 distal cuts of Therma-Ti Lite wires) were kept in self-closed sterilizing plastic bags.

### The control and the ex vivo groups

**Fig. 2:**
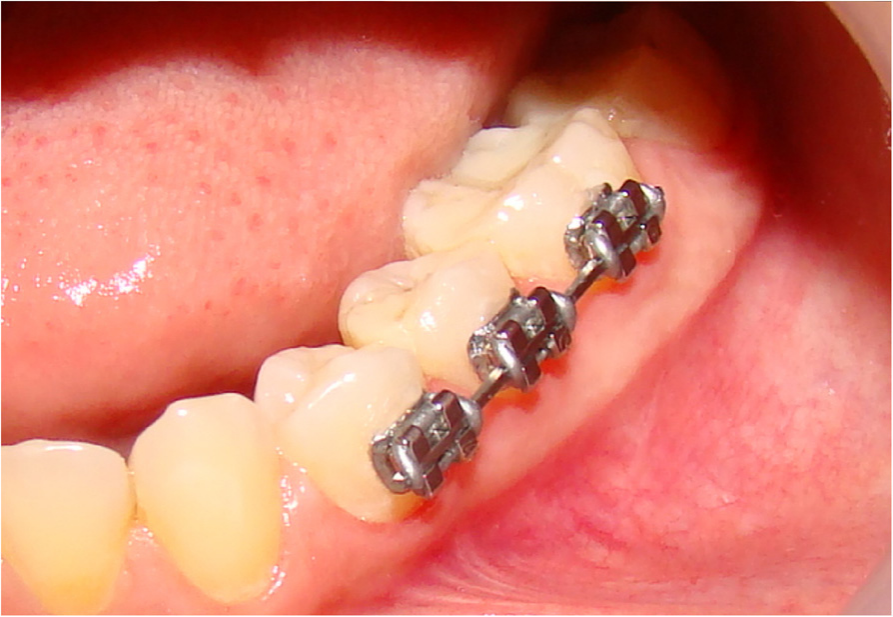
Experimental set after bonding

The control group consisted of 12 segments of NiTi Force I wires and 12 segments of Therma-Ti Lite wires that were left unused (“as received” state), whereas the ex vivo group consisted of 12 segments of NiTi Force I wires and 12 segments of Therma-Ti Lite wires. Each segment of the ex vivo group was passively tied using elastomeric ligatures (American Orthodontics, Sheboygan, WI, USA) to a triple set of the first lower premolar brackets, (Standard Edgewise, American Orthodontics, Sheboygan, WI, USA) extraorally. Then, sets were carefully bonded in each two lower semi arches of the volunteers from the first premolar to the first molar, as shown in Fig. 2. Each volunteer had two sets, one of them carrying superelastic NiTi segment, while the other one carries the heat-activated NiTi segment. The allocation of the side of the mouth receiving the superelastic or the heat-activated NiTi segments was based on computer-generated random sequence (Minitab® 15, Minitab Inc., State College, PA, USA).

### Volunteers’ recruitment

Prior to volunteers’ recruitment for this research project, ethical approval was obtained from the University of Al-Baath Dental School Local Ethics Committee (UBDS-1022-2012-PG). Consent forms were obtained for the volunteers following detailed explanation of the intended research work orally and in a written format.

The sample of possible candidates was derived from 186 fourth- and fifth-year dental students from the University of Al-Baath Dental School, Hama, Syria, depending on the following criteria: dental and skeletal class I malocclusion, age range between 21 and 24 years, healthy status with no syndromes or chronic diseases, good oral hygiene assessed by the plaque index and the gingival index of Löe and Silness [27], no significant dental caries causing significant destruction of one tooth surface, no metallic restorations, and if these had been observed, they were replaced with composite ones, no history of orthodontic treatments, good oral seal and no signs of oral breathing habits, no smoking or intake of liquid drugs and absence of chewing disorders (e.g., unilateral chewing).

Thirty-two students (12 males, 20 females) met the inclusion criteria, and only 27 (10 males, 17 females) of them accepted to participate in this research. Disproportionate stratified random sampling was employed to obtain a group of 12 volunteers (6 males, 6 females), and they were given instructions regarding maintaining good oral hygiene and keeping normal eating habits with no special dietary requirements.

After 30 days of wires’ placement in the oral environment, wires were collected and cleaned with 95 % ethanol to remove any precipitation, rinsed with ultrapure water to detach any loose bound deposits, then dried, and saved again in their self-closed sterilizing plastic bags.

The occlusal surface (6 mm from the end) of the wires’ segments (“as received” and “retrieved”) was tested to evaluate surface morphology and then immersed in artificial saliva to determine the amount of released nickel ions.

### Analyzing surface morphology

**Fig. 3:**
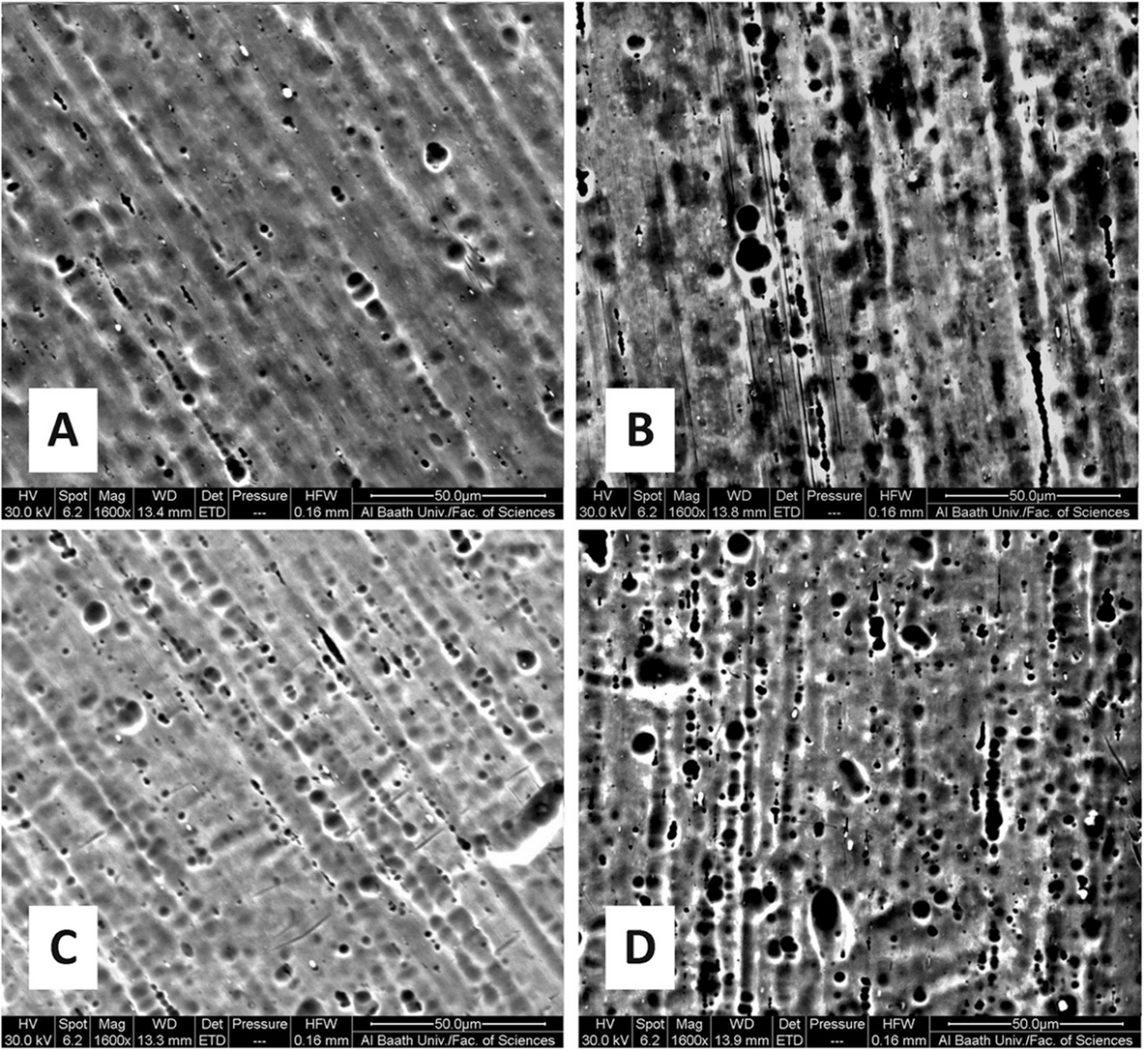
Scanning electron micrographs at ×1600 magnification of **A** as received superelastic NiTi wires, **B** retrieved superelastic NiTi wires, **C** as received heat-activated NiTi wires, and **D** retrieved heat-activated NiTi wires

The surface morphology and roughness of the wires were determined by using scanning electron microscopy (SEM; Quanta 200, FEI™, Hillsboro, OR, USA) and atomic force microscopy measurements (AFM; Nanosurf®, easyScan2, Liestal, Switzerland). Wires were set on the SEM chamber platform, and ×1600 magnification micrographs were obtained to give initial evaluation of wires’ surface roughness (Fig. 3).

**Fig. 4:**
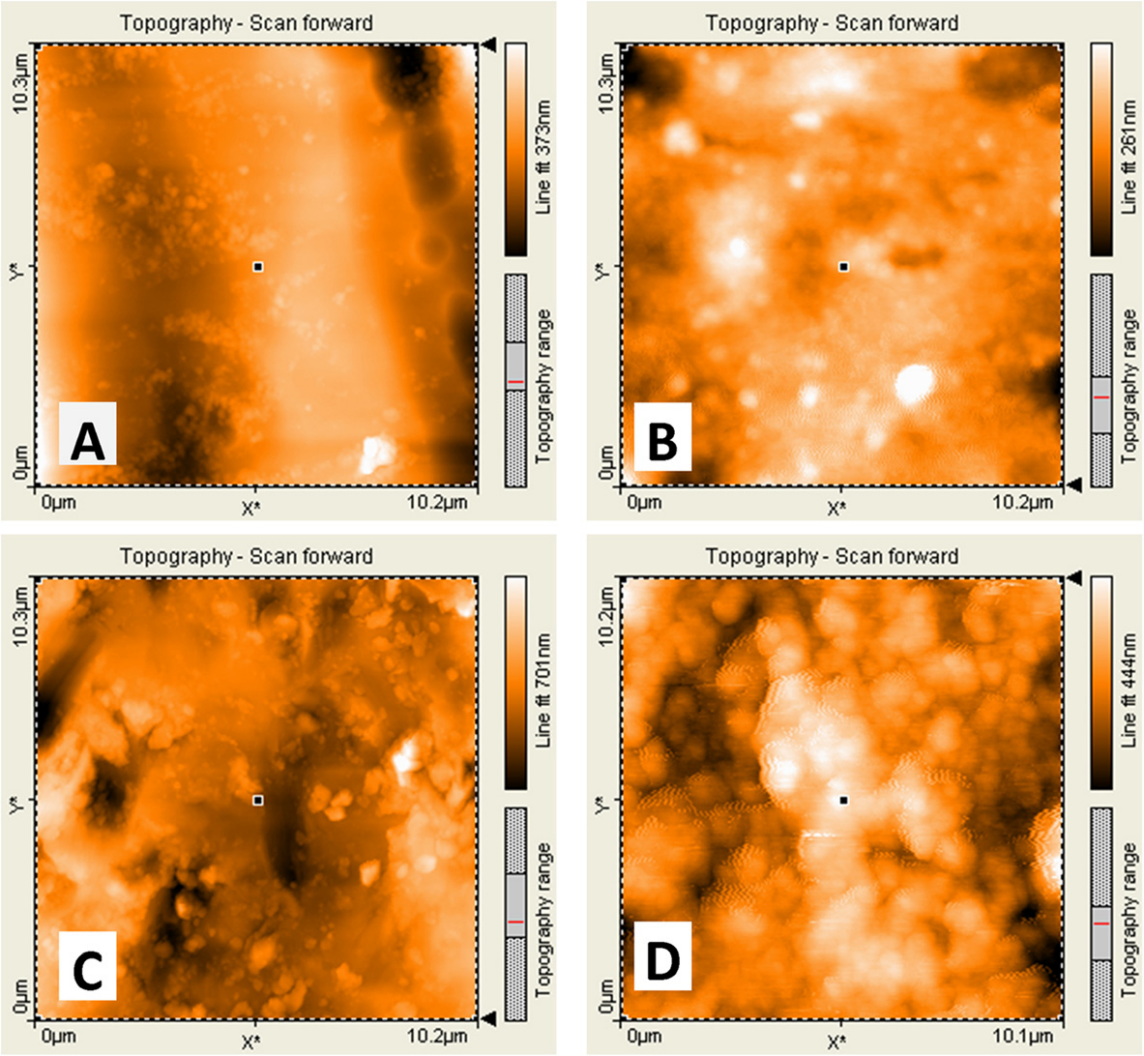
Atomic force two-dimensional micrographs (10 × 10 μm) of **A** as received superelastic NiTi wires, **B** retrieved superelastic NiTi wires, **C** as received heat-activated NiTi wires, and **D** retrieved heat-activated NiTi wires

**Fig. 5:**
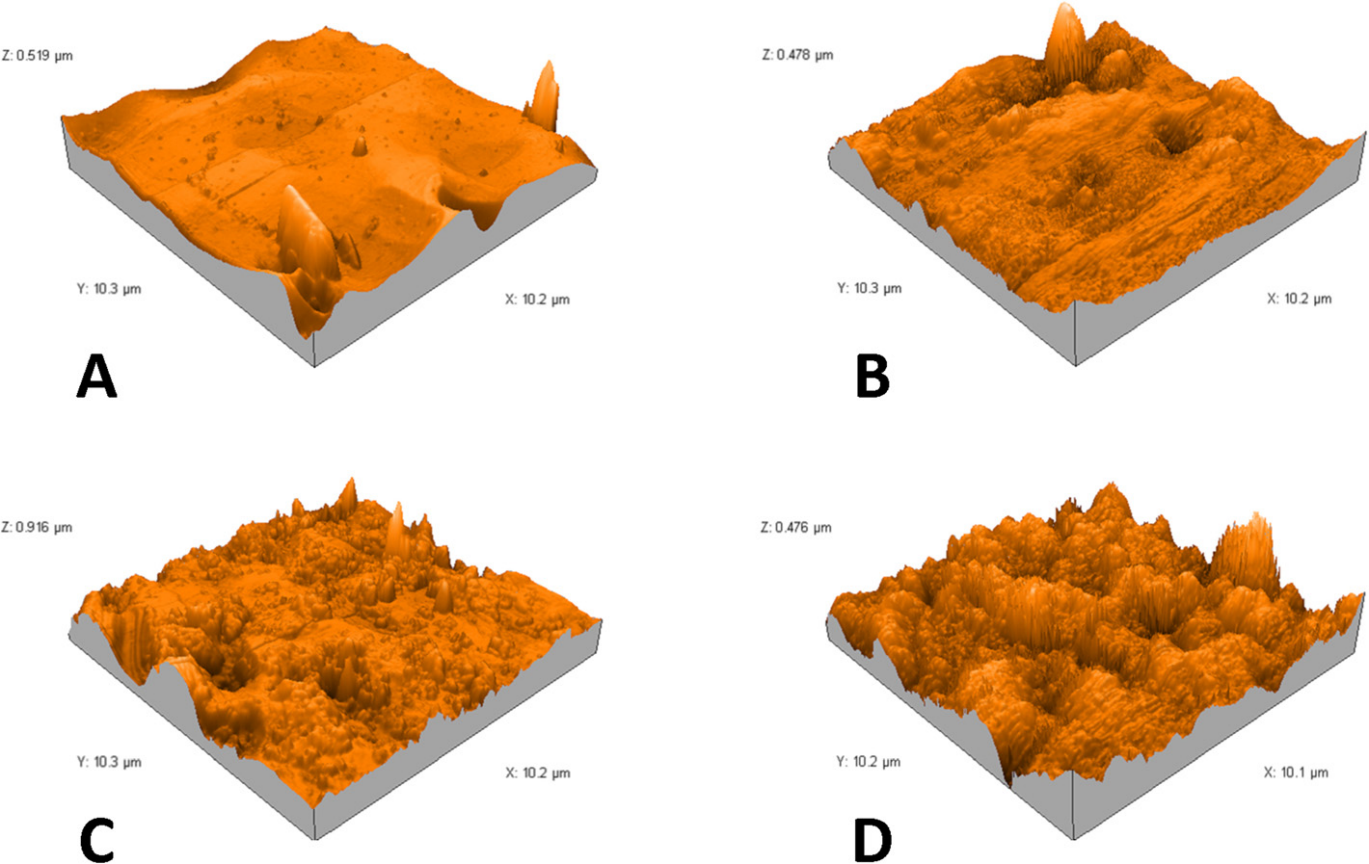
Atomic force three-dimensional reconstructed micrographs (10 × 10 μm) of **A** as received superelastic NiTi wires, **B** retrieved superelastic NiTi wires, **C** as received heat-activated NiTi wires, and **D** retrieved heat-activated NiTi wires

For further analysis of surface morphology, AFM measurements were performed in a tapping mode in air at room temperature ranged between 26° and 28°. Silicon cantilevers (Tap190 Al-G, NanoSensors™, Neuchatel, Switzerland) with 30-nm-thick aluminum reflex coating were used. According to the producer’s datasheet, the cantilever spring constant was in the range of 1.5–15 N/m and the resonance frequency was 15–500 kHz. The tip radius was less than 10 nm. The scan rate was set at 0.7 Hz, and the scanning size was 10 × 10 μm. The two-dimensional images were captured in the medium mode in a JPEG format (Fig. 4). Each image was processed using AFM Software (Nanosurf Report Expert 5.0 Nano Surf®, Liestal, Switzerland), and three-dimensional images (Fig. 5) were reconstructed using Nanosurf Report Expert 5.0 (Digital Surf, Nano Surf®, Liestal, Switzerland). The area of the roughness profile, Sa, is the parameter that was recorded to evaluate average surface roughness [24, 28, 29].

### Nickel ions’ release measurement

After analyzing surface morphology, both types of the wire samples (“as received” and “clinically exposed”) were placed in polypropylene vessels containing 2 mL of modified Fusayama artificial saliva with the following composition [19, 20]: NaCl (400 mg/L), KCl (400 mg/L), CaCl_2_·2H_2_O (795 mg/L), NaH_2_PO_4_·H_2_O (690 mg/L), KSCN (300 mg/L), Na_2_S·9H_2_O (5 mg/L), and urea (1000 mg/L) prepared using ultrapure water (WT 100 Millipore, Qamato, Japan), and then adjusted to a pH of 2 using HCl and maintained at 37 °C for 30 days.

Artificial saliva samples were treated with 5 % nitric acid [30], and standard solutions of nickel (Merck®, Germany) were prepared in the following concentrations: 0, 5, 10, and 25 particle per billion (ppb). Then, released nickel ions in artificial saliva were measured benefiting from standard solutions’ light absorption by using a graphite furnace atomic absorption spectrophotometer (GF-AAS) (AA-6800, Shimatzu®, Kyoto, Japan) that was programmed in advance according to the device manufacturer’s instructions to detect nickel ions depending on its wavelength (232.0 nm), and the following thermal program was successively applied on the samples: 120 °C for 20 s, 250 °C for 10 s, 800 °C for 10 s, 800 °C for 3 s, and 2500 °C for 3 s.

#### Statistical analysis

Minitab® 15 (Minitab Inc, State College, PA, USA) was used to perform the statistical analysis. With alpha set at 5 %, paired *t* tests were applied to evaluate intragroup changes (“as received” vs after “clinical exposure” in the same group), whereas two-sample *t* tests were employed to examine intergroup differences (superelastic vs heat-activated in the same wire state).

## Results

### Micromorphology

Scanning electron microscope (SEM) provided two-dimensional black and white micrographs of NiTi wires. Some SEM micrographs are shown in Fig. 3. SEM images demonstrated surface textures for each type of wires in the “as received” state, with an apparent greater surface roughness for the heat-activated wires. It was felt that more complicated surface textures were seen after clinical exposure for each of the two types of wires.

AFM analysis provided microscopic information on the surface structure that plot topographies representing the wires’ surface relief. Figures 4 and 5 show the 2D and 3D AFM microphotographs, respectively. The results of AFM surface analyses are given in Table 1.

**Table 1:** Average surface roughness of the superelastic NiTi group and the heat-activated NiTi group before (“as received”) and after (“retrieved”) clinical exposure

Average surface roughness, nm	Superelastic NiTi group (*n* = 24)			Heat-activated NiTi group (*n* = 24)			*P* value between the two groups
	Mean	SD	*P* value in the same group	Mean	SD	*P* value in the same group	
“as received”	55.73	14.04	<0.001	86.05	13.58	0.020	<0.001
“retrieved”	81.47	10.11		98.68	13.38		0.002
Difference between “as received” and “retrieved”	+25.74	10.50		+12.63	8.80		<0.001

*SD* standard deviation

In the control group “as received” superelastic wires showed less average surface roughness (55.73 nm) than heat-activated ones (86.05 nm) and the difference was statistically significant (*P* < 0.001). Following clinical exposure, the average surface roughness of superelastic and heat-activated wires significantly increased to become 81.47 (*P* < 0.001) and 98.68 nm (*P* = 0.020), respectively.

Though less rough surfaces were recorded for the superelastic wires after clinical exposure as compared with heat-activated wires (*P* = 0.002), the mean increase of roughness for the superelastic wires (+25.74 nm) was statistically greater than that of the heat-activated ones (+12.63 nm; *P* < 0.001).

### Nickel ions’ release

As shown in Table 2, the mean nickel release from “as received” heat-activated wires (8.36 ppb) was insignificantly less than that of the “as received” superelastic wires (7.92 ppb; *P* = 5.556). Following clinical exposure, less nickel ions were released from superelastic wires (5.81 ppb) compared to the same wires in the “as received” state (*P* = 0.005). A similar result was observed for the heat-activated wires (5.65 ppb; *P* = 0.008). Less nickel ions were released from the heat-activated wires after clinical exposure as compared with superelastic wires with no significant difference (*P* = 0.837).

**Table 2:** Average nickel ions’ release from the superelastic NiTi group and the heat-activated NiTi group before (“as received”) and after (“retrieved”) clinical exposure

Average nickel ions’ release, ppb	Superelastic NiTi group (*n* = 24)			Heat-activated NiTi group (*n* = 24)			*P* value between the two groups
	Mean	SD	*P* value in the same group	Mean	SD	*P* value in the same group	
“as received”	8.36	1.81	0.005	7.92	1.89	0.008	NS
“retrieved”	5.81	2.14		5.65	1.6		NS
Difference between “as received” and “retrieved”	−2.55	1.54		−2.27	1.10		NS

*SD* standard deviation, *NS* not significant

## Discussion

This study seems to be the first cross-over ex vivo study that compares superelastic and heat-activated NiTi wires apart from orthodontic forces. Wire segments were placed without inducing any actual tooth movement in a similar way to what was proposed by Marques et al. [12]. Neither volunteers nor researcher nor data analyzer knew the wire type in each side (triple blindness) in order to increase the validity of the conducted work.

Similar to previous reports, SEM micrographs revealed only descriptive characteristics of wires’ surfaces, whereas AFM images provided us with numeric values of the surface roughness and reconstructed three-dimensional images suitable for quantifying different aspects of micromorphology [23, 31, 32].

More rough texture was found in the non-used heat-activated NiTi wires when compared with superelastic ones. This could be attributed to the different procedures applied on heat-activated wires during manufacturing or tumbling and pickling stage. Other papers that reported the opposite finding [23, 24] compared the two types of wires but from different manufacturers with different chemical compositions.

Surface integrity of both types of NiTi wires were affected by oral environment conditions, i.e., temperature and pH variations, flora, masticatory and brushing forces, etc. The data from AFM showed an increase of 25.74 nm in superelastic NiTi wires’ roughness (i.e., 46.2 % increase) and an increase of 12.63 nm in heat-activated NiTi wires’ roughness (i.e., 14.7 % increase). This confirms the fact that crevice corrosion happened or calcified protein capsule was formed on the retrieved wires [6]. Other studies which demonstrated that there was no effect of clinical use on the NiTi roughness either used optical microscopy and/or SEM only [6, 11] or applied multiple disinfection procedures on wires before AFM measurements [25] that may have affected the accuracy of their results.

This study showed that heat-activated NiTi wires withstood oral conditions to a greater extent and the increase in their surface roughness was less than that recorded in the superelastic ones. This could be due to the fact that heat-activated NiTi wires used in this study had been originally fabricated to express all memory shape properties in 35 °C, whereas the superelastic archwires were not constructed to react favorably against different oral temperature variations. However, both types of wires maintained an acceptable degree of fine surface topography after clinical exposure based on the classification of Bourauel et al. [28], due to the fact that all Sa values were less than 200 nm at the final assessment.

Several factors that may have changed the current picture of results were not included in this study. The period of clinical use in our study was 30 days only, and this duration might be considered relatively short by some authors. The attached wires to braces were set neutrally and did not apply any active forces to the teeth. In addition, there were no big molecular changes on the archwire structure since superelastic wires were not exposed to stress and heat-activated wires were not exposed to cooling.

Though nickel is considered a toxic and cariogenic element, it is an essential component of orthodontic wires [15]. This made the biocompatibility of NiTi wires an important issue for researchers [11, 15, 33]. It is difficult to determine the exact amount of released nickel from NiTi wires in the oral cavity. So, in order to measure the potential release of nickel from NiTi wires in artificial saliva, a GF-AAS was used which is considered a reliable device in detecting released ions in a liquid sample by particle per billion [33–35].

An interaction was recorded between the nickel-titanium alloy and the surrounding environment (i.e., the artificial saliva) and both new superelastic and heat-activated NiTi wires released nickel ions. This is a support to the notion that this alloy is not an inert one [19]. Only tiny amounts of Ni ions were detected when evaluating unused superelastic and heat-activated NiTi wires (8.36 and 7.92 ppb, respectively). This may be because of the redundant Ni ions on the wires’ surfaces [19], those Ni ions unlinked to Ti in both types of wires (“used” and “unused”) could be easily released in artificial saliva. Kuhta et al. [36] found that superelastic NiTi wires released more Ni than heat-activated NiTi wires, but they studied wires from different manufacturers and immersed them in a different artificial saliva than that used in the current study.

After clinical exposure, GF-AAS detected less Ni ions released in artificial saliva from both types of wires with decrease of 2.55 ppb from superelastic NiTi wires that equals to −30.5 % and with decrease of 2.72 ppb from heat-activated NiTi wires that equals to −28.6 % that indicates a partial damage of the titanium oxide protecting layer causing the release of other alloy components [20]. This is the same result that Gil et al. [37] found after incurring NiTi wires to thermal sterilization treatments. They attributed the decline of released nickel in the reused archwires to depletion of nickel matrix caused by the precipitation of Ti_3_Ni_4_.

In order to understand this decrease in the Ni released ions from clinically exposed NiTi wires in the current study, it should be noted that the breaking of titanium oxide layer usually occurs in high rates in the first day of exposure to a corrosive conditions because of the attack from the oral environment conditions [11, 26]; while after 30 days of clinical exposure, a regrowth of passive film takes place [4, 26, 38]. Probably, the release of nickel ions from the surface oxide film on NiTi completed during clinical exposure and the composition of the oxide film changed with a less content of nickel. Since only small preexisted surface defects were detected on the wires’ surface, this may have helped in the regrowth process of the oxide layer to take place [18].

Fortunately, both types of NiTi wires released very small concentrations of Ni ions which were below the toxic levels [15, 39] that would impair the chemotaxis of leukocytes and motivate neutrophils to become aspherical and move slowly [40]. Ni ion concentrations were also below the critical value necessary to induce allergy [18, 41], and the current results go in line with the results of other researchers who have considered the NiTi alloy a safe and biocompatible material when used in the oral cavity [20, 41–43].

## Conclusions

This study showed that new heat-activated NiTi wires had more rough surfaces than superelastic NiTi wires, whereas both types released almost the same tiny amounts of Ni ions in the artificial saliva. NiTi wires’ surface roughness significantly increased after clinical exposure, whereas the amount of their released Ni ions in the artificial saliva decreased; an increase of surface roughness was observed to a greater extent in superelastic NiTi wires compared to the heat-activated ones, whereas the difference between them regarding the decrease of Ni ions’ release following oral exposure was not significant.
